# What interactions drive the salivary mucosal pellicle formation?

**DOI:** 10.1016/j.colsurfb.2014.05.020

**Published:** 2014-08-01

**Authors:** Hannah L. Gibbins, Gleb E. Yakubov, Gordon B. Proctor, Stephen Wilson, Guy H. Carpenter

**Affiliations:** a Salivary Research Unit, King's College London Dental Institute, London SE1 9RT, UK; b Australian Research Council Centre of Excellence in Plant Cell Walls, School of Chemical Engineering, The University of Queensland, Queensland 4072, Australia; c Unilever R&D Discover, Colworth Science Park, Sharnbrook MK44 1LQ, UK

**Keywords:** Saliva, Proteins, Pellicle, Hydrophobic, Transglutaminase

## Abstract

The bound salivary pellicle is essential for protection of both the enamel and mucosa in the oral cavity. The enamel pellicle formation is well characterised, however the mucosal pellicle proteins have only recently been clarified and what drives their formation is still unclear. The aim of this study was to examine the salivary pellicle on particles with different surface properties (hydrophobic or hydrophilic with a positive or negative charge), to determine a suitable model to mimic the mucosal pellicle. A secondary aim was to use the model to test how transglutaminase may alter pellicle formation. Particles were incubated with resting whole mouth saliva, parotid saliva and submandibular/sublingual saliva. Following incubation and two PBS and water washes bound salivary proteins were eluted with two concentrations of SDS, which were later analysed using SDS-PAGE and Western blotting. Experiments were repeated with purified transglutaminase to determine how this epithelial-derived enzyme may alter the bound pellicle. Protein pellicles varied according to the starting salivary composition and the particle chemistry. Amylase, the single most abundant protein in saliva, did not bind to any particle indicating specific protein binding. Most proteins bound through hydrophobic interactions and a few according to their charges. The hydrophobic surface most closely matched the known salivary mucosal pellicle by containing mucins, cystatin and statherin but an absence of amylase and proline-rich proteins. This surface was further used to examine the effect of added transglutaminase. At the concentrations used only statherin showed any evidence of crosslinking with itself or another saliva protein.

In conclusion, the formation of the salivary mucosal pellicle is probably mediated, at least in part, by hydrophobic interactions to the epithelial cell surface.

## Highlights

• Largely protein showed the ability to bind through hydrophobic interactions, yet some also bind according to their charges.
• The hydrophobic surfaces showed the closest match to the known bound mucosal pellicle.
• Salivary protein binding to particles was improved in some samples when incubated with transglutaminase.

## Graphical abstract

**Figure:**
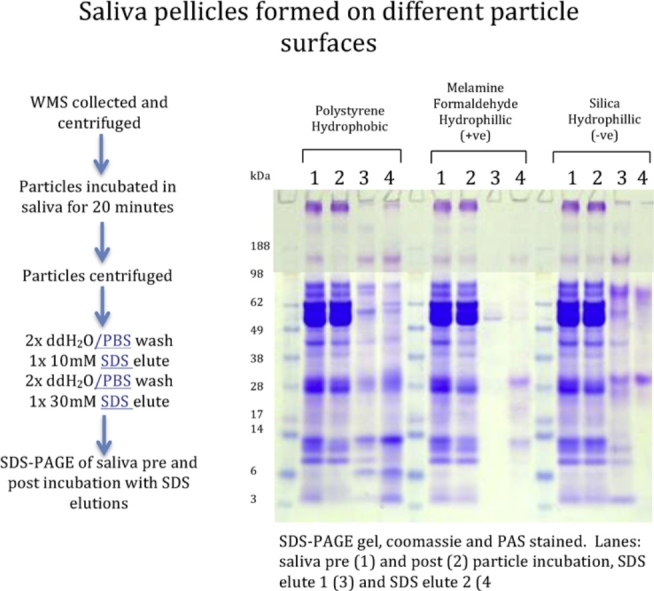

## 1 Introduction

### 1.1 The bound mucosal pellicle

The oral mucosa has to be extremely tough to withstand the extreme conditions it is exposed to, such as the abrasive action and temperature extremes associated with an extremely wide range of foods in the human diet. This concerns both modern human diet as well as the pre-historic one; from hot beverages and fire-cooked meats, down to sub-zero frozen desserts, and tough grasses and vegetables (including various tubers) that contain highly abrasive silica particles (phytoliths) [1]. The oral cavity has two lines of defence; firstly, the parts of oral mucosa that are under direct action of mechanical forces such as the hard palate developed into mechanically tougher keratinised tissues, designed to protect the underlying cells from damage [2]. Secondly, the harsh mechanical environment of the oral cavity is tempered by the lubricating effect of the salivary pellicle that protects both tooth enamel and soft tissue [3–5], including softer non-keratinised oral surfaces such as for example buccal mucosa. The bound mucosal pellicle is a supra-molecular film with a complex architecture that comprises several structural layers. It comprises a complex of many salivary proteins including: sIgA, MUC5B, MUC7, carbonic anhydrase VI (CAVI) and cystatin S [6,7]. Salivary mucins, MUC5B and MUC7 are key for providing layer protection and lubrication due to their high molecular weight and high level of hydration which is due to the presence of highly glycosylated regions. Both type of salivary mucins are found to be strongly retained on the buccal cell surfaces [8,9], while within tooth enamel pellicle the mucin composition is dominated by MUC5B [6]. The self-assembly process of salivary proteins varies greatly depending on the type of oral surfaces, with variations in composition, protein content, thickness and the rate of replenishment. The key element of the assembly process is the formation of a tightly bound layer that ensures adhesion of the pellicle and also acts as a template for further protein/mucin assembly.

### 1.2 Formation of the bound mucosal pellicle

Adsorption of individual salivary proteins and whole saliva have been widely studied on different surfaces. Hydroxyapatite (HAP) has largely been studied as a model for the enamel pellicle [10]. Tooth enamel, being a mineral surface, has a number of distinct features. Thus, the enamel pellicle contains significant levels of statherin, proline-rich proteins, and CAVI, essential for re-mineralisation/demineralisation of the enamel [4,5]. Statherin has a particular affinity to the hydroxyapatite surfaces due to the presence of Ca^2+^ binding domains. By contrast, it has poor retention on the buccal cell surface [7], and hence is considered to be a specific constituent of the enamel pellicle [11]. Statherin, PRP-1 and PRP-3 have all shown the ability to bind to both hydrophobic and hydrophilic surfaces, but to a much lower extent on the later with exception of PRP-1, due to its lower negative net charge [12].

MUC5B contains both hydrophilic heavily glycosylated domains, and hydrophobic domains located within non-glycosylated areas [13]. MUC5B has also been shown to have stronger adsorption to hydrophobic surfaces, as opposed to hydrophilic, leading to higher adsorbed mass and slower desorption times [12,14]. The addition of calcium has also been shown to facilitate MUC5B deposition through promoting protein cross-links [15]. Unlike MUC5B, MUC7 has much smaller molecular weight (250 kDa versus over 2000 kDa for MUC5B) and comprises a single glycosylated region surrounded by relatively small non-glycosylated domains [16]. Due to a larger relative size of the glycosylated domains, MUC7 has much higher levels of hydration which effects weaker adsorption. However, MUC7 has high propensity to self-associate which can counteract its high solubility and increase incorporation into the pellicle due to physical entanglements and formation of complexes with lower molecular weight proteins such as IgA [17,18].

The process of salivary protein adsorption and binding onto surfaces is complex due to the number of proteins present, varying protein size and individual protein concentration. This complex process is governed by a finely tuned accord of electrostatic and hydrophobic forces, hydrogen bonds, as well as specific binding interactions and chemical cross-linking. Many factors can influence salivary film formation, for example, ionic composition can have a significant influence on pellicle development, through increased/decreased level of electrostatic interaction and protein cross-linking [15]. Despite shear multitude of interaction mechanisms, certain common interaction patterns did emerged. Thus, a number of research groups investigated the surface deposition/adsorption of saliva; it has been established that salivary proteins demonstrate much higher affinity to hydrophobic surfaces [14,19–22]. This goes in line with the fact that the bare oral mucosa is a largely hydrophobic surface, which becomes more hydrophilic as proteinaceous layer builds up [23]. Proteinaceous layers can be formed on hydrophobic surfaces from whole mouth saliva (WMS), parotid saliva (PS) and submandibular/sublingual saliva (SMSL). By contrast, on hydrophilic surfaces the deposited amounts are lower, which is particularly striking for PS that does not form a stable film on hydrophilic surfaces [24,25], which can be associated with the high concentration of salivary amylase in PS secretions. We note that most salivary proteins participate in pellicle formation. However there are notable exceptions, thus on oral epithelial cells amylase, one of the most abundant salivary proteins, shows minimal binding within the bound mucosal pellicle [7].

Alternative explanations suggested associate the degree of deposition with the presence of proteins such as transglutaminase (TGM) that can aid in protein cross-linking thereby facilitating pellicle formation [3,5,26]. Statherin and PRP-1 are among those shown to crosslink due the presence of TGM [27,28]. TGM3 has been confirmed to be present in the mucosal pellicle in both pro-enzyme form and in its active form [7]. However, the lack of statherin and PRPs in pellicles formed on various artificial substrates suggests that the role of TGM in the pellicle development is not always critical.

### 1.3 Aims

The aim of this study was to elucidate mechanisms of salivary binding by exploring which salivary proteins bind to hydrophobic, hydrophilic positive and hydrophilic negative charged particles using un-stimulated whole mouth saliva (UWMS), PS and SMSL. How strongly proteins bind and how well retained proteins are will be compared between saliva types. The role of TGM will also be investigated to see if this improves protein retention and aids in pellicle development. It is predicted that a set of particles with different surface chemistries will allow a more in-depth mechanistic insights that otherwise can be complicated by a complex nature of real biological surfaces. It will also mimic the chemically diverse spectrum of surfaces in the oral cavity and provide a suitable material to study mucosal pellicle development. Finally, if a suitable model is found, it could be used for further studies of the mucosal pellicle. This capability aspect of this work is of particular interest since enamel and soft tissue (e.g. buccal) mucosa surfaces require laborious sourcing, as well as raise considerable ethical considerations with studies in vivo.

## 2 Methods

### 2.1 Saliva collection

UWMS, PS and SMSL were collected from two volunteers, who refrained from eating, drinking and using mouth-cleaning products for 1 h prior to collection. UWMS was collected by drooling into universal tubes until 2 ml+ had been collected. PS was collected using a Lashley cup attached to one of the parotid glands and a citrus sweet was used to stimulate saliva production until 2 ml+. SMSL was also collected in a universal tube by blocking off the parotid glands with dental roll, which absorbs any secretion. A mucus-specimen trap was then used to draw up SMSL, which was allowed to pool in the bottom of the mouth following chewing stimulation. All saliva was collected fresh for each experiment and used immediately for incubation on the different particle types. UWMS was centrifuged before use at 5000 RPM for 5 min.

### 2.2 Particle preparation and saliva incubation

Different particles were selected for their different surface types: polystyrene (PSt) (hydrophobic) (Bangs Labs, Fisher, IN, USA), melamine formaldehyde (MF) (hydrophilic positive) (microParticles GmbH, Berlin, Germany) and silica (Si) (hydrophilic negative) (Kisker Biotech GmbH & Co. KG, Steinfurt, Germany). The particles were all stored in a liquid suspension and it was calculated that 100 μl, 200 μl and 400 μl of each suspension was need respectively to have approximately 405 cm^2^ surface area, which would provide a surface area large enough for 1 ml of saliva to form a 7 nm thick film. All particle suspensions were topped up to 1 ml with PBS and water (1:1) (WPBS), which is a similar ionic concentration to saliva, and then centrifuged for 20 min at 10,000 rpm, 4000 rpm and 2000 rpm respectively, which provided a pre-wash prior to saliva incubation.

All particle types were incubated with 1 ml of UWMS, PS or SMSL saliva for 20 min, a time known to be long enough to form an in vitro pellicle [22], whilst being turned constantly at room temperature and then centrifuged at the previous speeds for 10 min. This was followed by 2 washes with 1 ml of WPBS, diluted to match ionic concentration of saliva, and centrifugation as before to remove residual saliva. MF particles were then centrifuged at 2000 rpm, like the Si particles, whilst PSt particles were still centrifuged at 10,000 rpm. A 100 μl of 10 mM SDS was then added for 12 min to elute proteins, followed by centrifugation, 2 more 1 ml WPBS washes and a final elution in 100 μl 30 mM SDS for 2 h at 80 °C in a heat block. Later a 100 μl boiling step was added, using water containing 50 mM DTT (Invitrogen, Paisley, UK) and LDS (Invitrogen) diluted at a ratio of 1:4 to determine the presence of any residual proteins on the surfaces of the particles.

### 2.3 Protein detection

SDS-PAGE was performed on all saliva samples, before and after incubation and on all SDS washes. All samples were prepared with 0.5 M DTT reducing buffer (1:10) (Invitrogen) and LDS sample buffer (1:4) (Invitrogen) and boiled for 3 min. 15 μl of sample was then loaded onto a lane of a 4–12% Bis-Tris gel (Invitrogen) and all samples were run according to manufacturer's instructions in MES-SDS running buffer. Following this, proteins were visualised using Coomassie brilliant blue R250 stain (Sigma, Dorset, UK), de-staining was completed in 10% acetic acid. After being photographed gels were fixed in 25% methanol and 10% acetic acid for 1 h followed by 20 min in a ddH_2_O wash. The gel was then oxidised in 2% periodic acid (Sigma) for 15 min followed by 2 more 2 min ddH_2_O washes. Schiff reagent (VWR, Lutterworth, UK) was then added for completion of a periodic acid Schiff stain (PAS), which indicates the presence of glycoproteins including MUC5B and MUC7.

Western blotting was used to visualise specific proteins. Western blotting was completed following electrophoresis, transferring proteins onto a nitrocellulose membrane, according to manufacturer's instructions. Membranes were blocked in TBS with 1% Tween added (TTBS) or TTBS with 2% milk powder (Marvel, Spalding, UK). Membranes were then incubated in primary antibodies: cystatin S (1:2000) (R and D Systems, Abingdon, UK), MUC5B (1:100), MUC7 (1:100), statherin (1:1000) and secretory component (1:500) (Dako, Ely, UK). This was followed by 3 × 15 min TTBS washes, incubation with the desired secondary antibody and then 3 final TTBS washes before development with the a chemiluminescent substrate, 90 mM coumaric acid and 250 mM luminol with H_2_O_2_ (Sigma). The membrane was then left to expose onto photographic film, developed and then fixed in the dark, followed by a water wash.

Maclura pomifera agglutinin (MPA) lectin (Vector Laboratories Ltd., Peterborough, UK) was also used to visualise proteins containing a Galβ,1-3GalNAc group. Several salivary proteins can be picked up with this lectin including the mucins, glycosylated PRPs and salivary agglutinin. This biotinylated lectin was used at 1 μg/ml followed an ABC kit (Vector Laboratories Ltd.) for 30 min and then binding detected by chemiluminescent detection as above.

### 2.4 Transglutaminase (TGM) cross-linking test

10 μl of 10 U/ml TGM (Sigma) was added to 1 ml WMS and PS obtained from 4 volunteers 20 min prior to particle incubation and compared to the saliva binding alone. Binding experiments were completed as previously described but WPBS washes 3 and 4 were omitted as minimal proteins were removed in the previous experiments with these washes. Particles were also boiled in 100 μl DTT, LDS and water to see if more protein remained on particles surfaces after all the other washes were completed. Samples were then processed in the same manner as the earlier ones.

The WMS and PS of these volunteers was also used in a TGM assay to determine whether cross-linking of the saliva could be seen visually using gel electrophoresis run under non-reducing conditions (no DTT and no boiling of the samples). 100 μl saliva was incubated for 20 min with 1 μl or 10 μl of 10 U/ml TGM and then samples prepared immediately following the addition of a protease inhibitor cocktail (Calbiochem, Merck, Darmstadt, Germany) at a concentration of 1:100, for SDS-PAGE. Western blotting was also completed to test for any changes in statherin in PS samples, using the method as described previously.

## 3 Results

### 3.1 Which proteins bind to the different surface types?

**Fig. 1:**
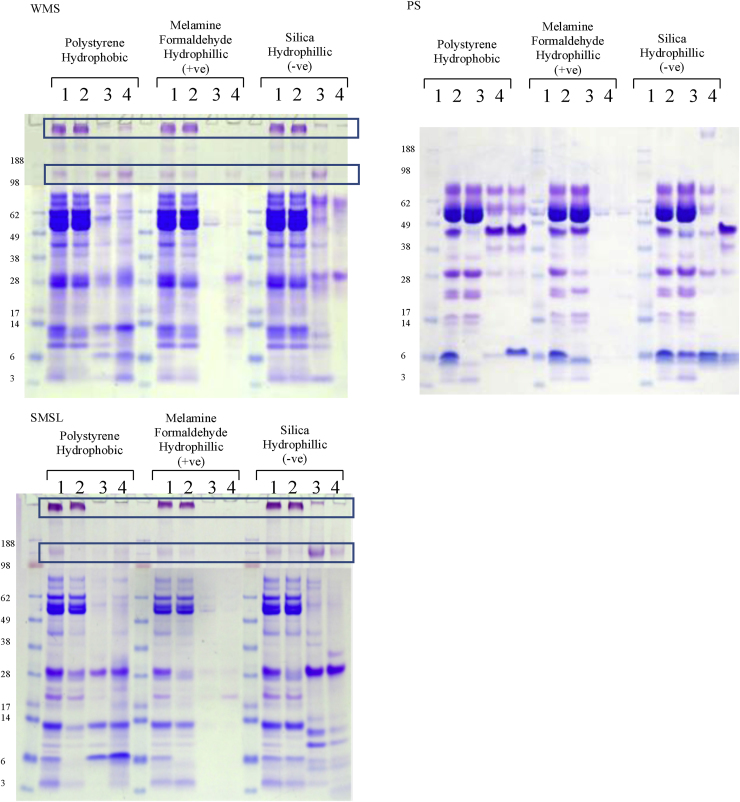
CBB and PAS stained gels (WMS and SMSL only), following gel electrophoresis of reduced samples. Lanes: saliva pre (1) and post (2) particle incubation, SDS elute 1 (3) and SDS elute 2 (4). PAS stained of WMS and SMSL have been merged with the CBB gels. Lanes 3 and 4 are concentrated 10× to allow an equivalent volume to be loaded. Salivary mucins are highlighted in the boxes.

**Fig. 2:**
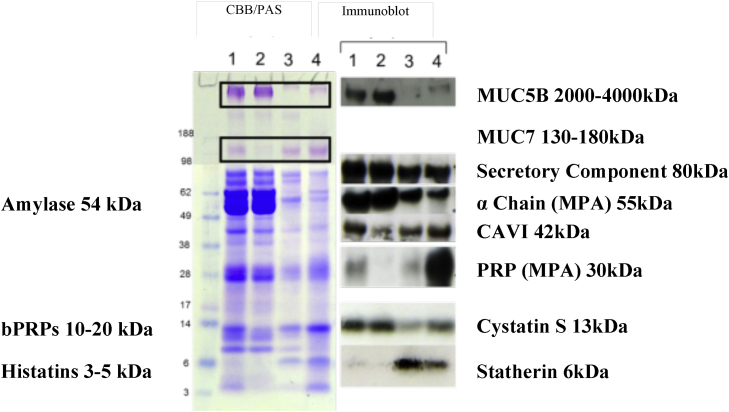
Protein confirmation on hydrophobic particles using Western blotting. Lanes: WMS pre (1) and post (2) particle incubation, SDS elute 1 (3) and SDS elute 2 (4). Salivary mucins, MUC5B and MUC7 are highlighted in boxes on CBB/PAS stained gel.

A number of salivary proteins bound to all particle types, see Fig. 1, most bound to at least one particle and a few highly abundant proteins did not bind at all. For example, MUC5B and amylase show minimal binding to all particle types from all saliva samples. Table 1 summarises the relative abundance of bound salivary proteins in the pellicle estimated based on CBB and PAS staining characteristics and immunoblotting (see also Fig. 2). Only a few proteins were found to bind all three particle types; these are MUC7, secretory component, IgA and aPRP (28 kDa), and to some extent cystatin S and CA VI. In some cases IgA and secretory component may bind together as part of the sIgA complex. Based on Fig. 1 the positively charged hydrophilic particles appear to have the least number of proteins binding to them, as only some aPRPs, cystatin S and relatively small amounts of MUC7 and IgA are present. This result is somewhat counter-intuitive since p*K*_a_ of the majority of salivary proteins (except lactoferrin and lysozyme) is below salivary pH [29] which is normally between 6.5 and 7.5. This means that salivary proteins bare largely a negative charge. The result can be explained if we suggest that binding to positively charged particles is so strong that binding kinetics favours very quick adsorption of highly negative species (possibly phosphorylated) such as aPRPs. This film can be very thin and hence the total amount of proteins adsorbed is very small. Also, due to fast kinetics, the composition is confined to a selected protein species that are either highly charged or present in saliva in a relatively high abundance. By contrast, for hydrophobic and negatively charged hydrophilic surfaces the adsorption process is driven by hydrophobic and van-der-Waals interactions, and hence may be slower. This slower kinetics may facilitate formation of a thicker and more complex (in terms of composition) proteinaceous film.

**Fig. 3:**
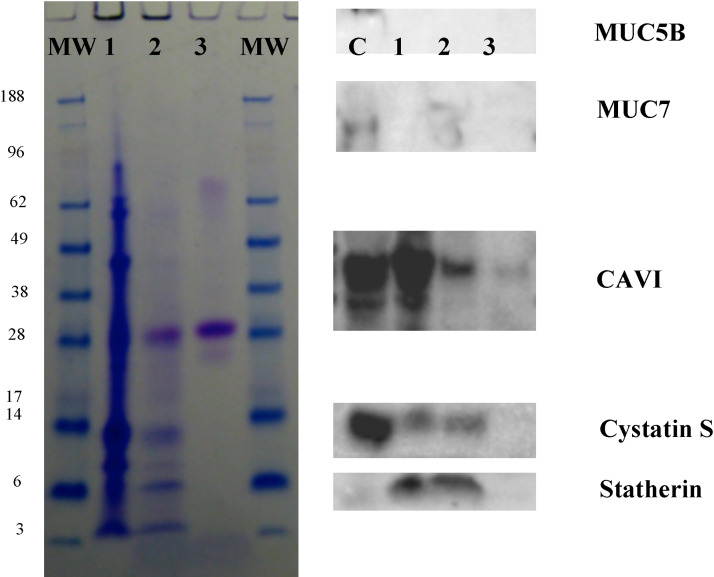
Gel electrophoresis and Western blotting of boiled elution (DTT/LDS) (100 μl) of particles, these followed SDS elutions to detect any remaining proteins on the particle surfaces. Lanes: boiled elution of PSt (1), boiled elution of MF (2), boiled elution of Si (3). C (blot only) WMS control.

Fig. 3 shows boiled washes, with DTT and LDS, completed after the WPBS washes and SDS elutions on WMS incubated particles. This experiment assessed whether there were any salivary proteins left on the particle surfaces. Indeed, as seen in Fig. 3 there are only a few proteins that are still adhered to particle surfaces. Neither mucins were retained on any particles types. Statherin was still retained on PSt and MF, despite being partially removed by SDS. Proteins including cystatin S and CAVI were still adhered to a certain extent on all particle types despite some removal in SDS washes. The gel electrophoresis also shows there was still very strong adherence of the 28 kDa aPRP on both the MF and Si particles. There were no proteins that showed a decrease between Fig. 1 lanes 1 and 2, that do not appear in lanes 3, 4 (or the boiled samples in Fig. 3) which indicates that most bound proteins were eluted with the methods used.

### 3.2 Transglutaminase and its effect on salivary pellicle development

**Fig. 4:**
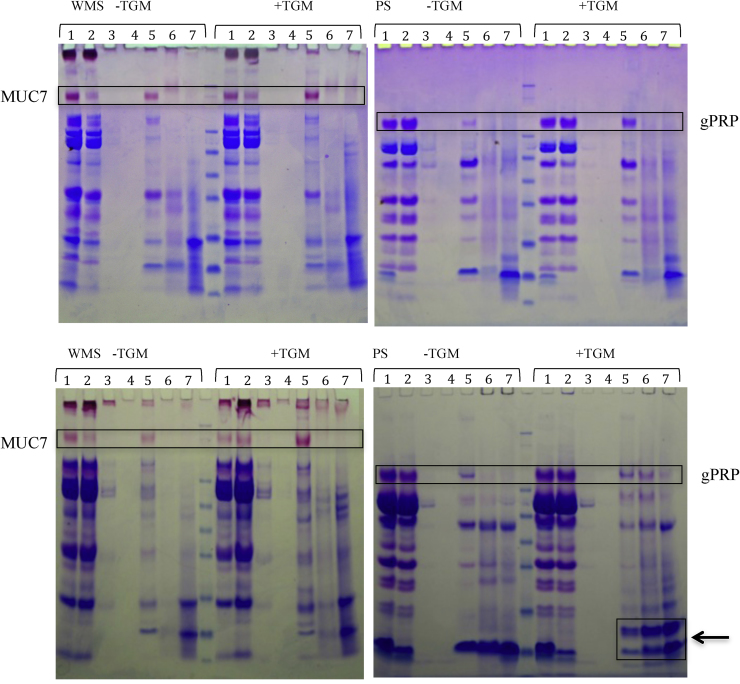
Gels following CBB and PAS staining (WMS only) of WMS and PS pellicle formations on hydrophobic particles from different subjects. Lanes: saliva pre (1), saliva post (2), water wash 1 (3), water wash 2 (4), SDS 1 (5), SDS 2 (6) and boiled DTT, LDS and water (7). Boxes highlight clear differences in binding ± TGM. The box highlighted by the arrow indicates an effect only seen in 50% of the samples. It shows what appear to be a possible cross-linked proteins, perhaps statherin and histatin bound higher due to crosslinking.

Pellicle forming experiments on hydrophobic particles with and without added TGM yielded volunteer dependant results. Binding of most proteins in both WMS and PS were not affected by the presence of TGM. However, some proteins bound to particles at a higher concentration when TGM was added to the saliva, as shown in Fig. 4. In WMS samples MUC7 binding to the hydrophobic particles was greater with TGM present and in parotid saliva glycosylated PRP (gPRP) also showed the same pattern, as highlighted in the boxes.

A curious effect was noticed in 3 out of the 4 PS samples with TGM binding was the presence of extra bands at approximately 10 kDa, as indicated by the arrow and box in Fig. 4. Despite apparently more statherin binding due to a greater reduction in the saliva post-incubation samples (lane 2) this is not shown by more statherin (at 7 kDa) in the SDS elutes and boiled samples. However the presence of a higher band at 10 kDa may be representative of statherin cross-linked with another protein, potentially one of the histatins that normally run between 3 and 5 kDa as this could match the estimated molecular weight, or even itself. Further analysis is required to establish the identity and mechanism of this novel protein complex.

**Fig. 5:**
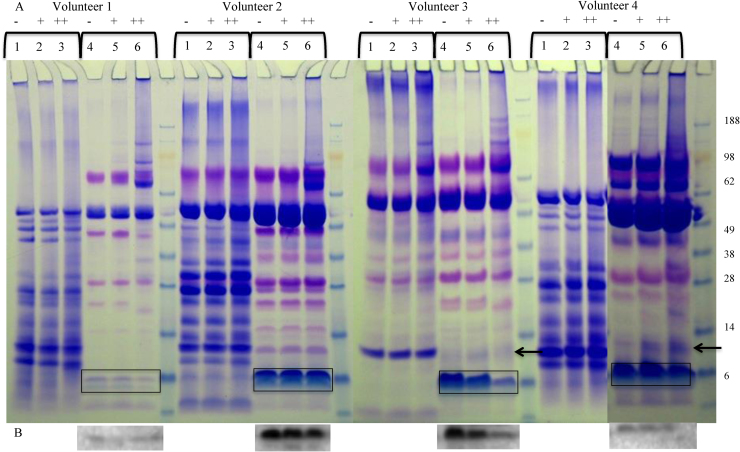
(A) SDS-PAGE of 4 volunteers WMS (Lanes 1–3) and PS (Lanes 4–6) ± TGM at 0.1 U/ml (Lanes 2 and 5) and 1 U/ml (Lanes 3 and 6) following 20 min incubation. Samples volumes equalised with water. Boxes indicate statherin on gels. (B) Statherin blots of PS samples. Arrow indicates the development of a new band.

As there were mixed results between volunteers for the pellicle formation ± TGM, cross-linking of salivary proteins with TGM alone (no particles) was examined on a non-reduced gel, see Fig. 5. PAS stained gels showed no difference with regards to the salivary mucins cross-linking in the presence of TGM, despite improved MUC7 binding in the presence of TGM (data not shown). Cross-linking of proteins was seen at the higher concentrations of the TGM incubations as indicated by the heavier staining at the top half of the gel. However, obvious cross-linking of specific proteins was not clear in all samples. Volunteers 1 and 3 showed a clear reduction of statherin and histatin bands in PS as highlighted in Fig. 5, which would be the proteins that may match with the development of a new band at approximately 10 kDa due to cross-linking binding to particles. In volunteers 3 and 4 at approximately 10 kDa we saw the development of a protein band in the PS samples as indicated by the arrow. In volunteer 4 the decrease in statherin was not visible due to its high concentration in this sample, this was later confirmed by Western blotting, see Fig. 5b. Changes in PRP bands can also be seen, particularly at higher concentrations of TGM in PS, suggesting they may be involved in protein cross-linking through TGM which could alter pellicle formation and development.

## 4 Discussion

### 4.1 Hydrophobic versus hydrophilic binding

Saliva produced by all three major salivary glands: parotid, submandibular and sublingual, clearly has the ability to form protein bound pellicles on both hydrophobic and hydrophilic surfaces despite the variation in salivary composition. However, there are significant differences in the amount of protein binding to the different surface types. Our work coincides with data collected by Lindh et al. [12,30], which showed lower levels of salivary protein binding to hydrophilic surfaces. In particular the hydrophilic positive charged surface appears to bind the lowest variety of proteins, although elutions and washes may not completely remove all of the bound proteins. However, protein recovery was checked and most proteins appear to be accounted for. However, those still present on the hydrophilic positive surface after the main experimental washes appear to be very small (<30 kDa), including bPRPs, 28 kDa aPRP, cystatin S and statherin, most of which are only removed by the boiling in DTT and LDS suggesting a very strong interaction. All of these proteins decreased in post-incubation saliva samples, but were not present in the SDS elutions. This strong binding is likely to be due to the greater number of negatively charged residues within the proteins compared to number of positive residues [10,31]. However, aPRPs are negatively charged in saliva, having a p*I* of 4 [32], so their unstructured nature is likely to explain their ability to bind to positively charged and negatively charged surfaces [33], or protein cross-linking/interactions may be occurring. aPRP also bound to hydrophobic particles suggesting hydrophobic interactions with surfaces too. Being smaller in molecular weight in comparison to many salivary proteins, their size also allows the proteins to adhere more quickly to the hydrophilic positive particles and perhaps prevents the larger proteins binding. However, Lindh et al. have highlighted that salivary proteins when bound alone have shown much less binding to hydrophilic surfaces, which eliminates any competition from other salivary proteins [12,30].

The hydrophilic negatively charged particles were also found to bind several proteins, which include the higher molecular weight PRPs, including the gPRPS, as well as the salivary mucin MUC7. However, these were poorly retained on the surfaces and only the 28 kDa aPRP was retained on the particle surface after the two SDS elutions. As mentioned previously, the ability of salivary PRPs to bind to both hydrophilic positive and negative particles may be due to their intrinsically unstructured nature [33], which may lead to more charged protein residues being exposed to bind to multiple surface types.

### 4.2 Binding changes depending on protein source

PS binding to the hydrophobic particles resulted in the gPRPs and aPRPs showing the ability to bind to the hydrophobic surfaces, where proline residues are able to provide binding sites [33]. However, when UWMS is bound, binding of these proteins is reduced. This could indicate the competition between these PRPs and other SMSL proteins or highlight the effect the different proteins have on each other, potentially indicating the importance of protein cross-linking within the pellicle. For example, mucins showed reduced binding in SMSL on hydrophobic particles compared to UWMS.

Of those tested, the hydrophobic particles appear to be the only surface the MUC5B binds to, but at a very low level. This heavily glycosylated protein is thought to be an essential part of the enamel and mucosal pellicle [7] and with its gel forming properties is thought to be essential for lubrication in the oral cavity [15]. MUC5B has both hydrophilic domains and hydrophobic domain patches [12,30], however its hydrophobic domains are within the non-glycosylated region and it is possibly covalently bound lipids from saliva that contribute to its hydrophobic nature [34]. Its low level of binding may also be due to the lack of membrane bound MUC1 on the particle surfaces [35,36] which could be essential in the binding of MUC5B to the mucosa in the oral cavity and development of the pellicle.

MUC5B is also known to exist in several different glycoforms [37,38], which may alter its binding properties. As MUC5B showed no binding from SMSL, this may indicate a more neutral self-assembled mucin structure, essential for the viscoelastic properties of saliva [39]. When present in UWMS within the soluble gel phase, the mucin may become more charged and binding levels thus improve [37,40].

MUC7 however appears to bind to all surface types, perhaps due to lower levels of glycosylation [16]. It can also form cross-links with other salivary proteins such as sIgA and lactoferrin, which may improve their incorporation into the pellicle layer [18,41], as well as its own incorporation. This may be evident if we consider that IgA and MUC7 are among only a few proteins that bind to all surfaces (see Table 1) and that the IgA binding from PS is reduced, i.e. when not in the presence of MUC7.

The overall pattern of protein binding to particles suggests that most are binding according to their charge or hydrophobic interactions. Small proteins including, statherin and cystatin S, also showed strong adherence perhaps due to their size and ability to bind more quickly than the large globular mucins. Amylase appears to be the anomaly; despite it being the most abundant protein, its lack of binding suggests it is not easily retained by surfaces and perhaps needs to be involved in protein cross-linking within the pellicle or that an “amylase receptor” may be required. Both sIgA and amylase have a relatively neutral charged [42] in saliva and one could assume that both would bind well through hydrophobic interactions, yet amylase is poorly bound in comparison to IgA and perhaps lacks interactions with other proteins which could be essential for pellicle formation. Another factor for consideration is the fact that 25% of secreted amylase is glycosylated, within the two main amylase forms at 56 and 59 kDa, with many different isoforms [43]. This could result in hydrophobic regions and charged resides being masked within the molecule, impairing amylase adsorption to a surface.

### 4.3 Does any particle type mimic the oral mucosa?

With regards to which particle surface most represents the pellicle in the mouth, we would suggest the hydrophobic particles provide the best model out of the three surface types, given that it binds most salivary proteins. The hydrophobic particle pellicle is also the closest match to the oral epithelial cell pellicle determined from our previous work [7]. It is likely that the oral mucosa surface is initially hydrophobic, matching the mucosal pellicle on hydrophobic particles, but becomes hydrophilic as a result of protein adsorption [23]. A study by van der Mei et al., has proposed a similar mechanism whereby salivary pellicles formed on enamel were initially polar but following absorption of salivary proteins became more apolar [44].

The hydrophobic particles also appear to retain the most salivary proteins after the two SDS elutions, which may indicate that this hydrophobic binding is a more important interaction in the oral cavity with regards to pellicle formation. SDS will make proteins negatively charged; protein cross-linking would then prevent hydrophobic bound proteins being removed from particles, suggesting its crucial role pellicle development.

### 4.4 Effects of TGM on pellicle development

The hydrophobic particles were used as a model to study how TGM alters pellicle development. TGM showed an ability to alter the pellicle formed on the hydrophobic particles. In general there was an increase in proteins bound, seen through greater amounts of protein in the SDS elutes and boil washes (as shown in Fig. 4). Particular experiments showed higher levels of gPRP, aPRP, cystatin S and statherin. In SDS eluted samples of some subjects, there was the presence of a new protein at approximately 10 kDa in the TGM samples. This is likely to represent a cross-linked unit of two proteins, mediated through the enzymatic action of TGM. Statherin, histatins and aPRPs have been confirmed as proteins that can be crosslinked by TGM [27]. This 10 kDa may represent statherin and histatin crosslinked based on their molecular weight, or even a mix of histatins 1, 3 and 5 [45]. This data would match the saliva assays, where Fig. 5 shows a decrease in statherin/histatin following incubation of saliva and in some cases a development of a band at 10 kDa, which may represent statherin/histatin cross-linked. The saliva sample from volunteer 2, where this effect was not seen, may not have the protein that statherin cross-links with, resulting in no change seen with TGM with regards to statherin cross-linking.

The presence of TGM also leads to greater adherence of MUC7 in WMS pellicle formation, as demonstrated in Fig. 4. This was unexpected, as this effect of TGM on MUC7 has not been observed previously and it was not shown to be cross-linked in the TGM activity assay. However, this may be due to an indirect effect, such as improved binding of a protein with which MUC7 forms cross-links. As several PRPs, statherin and possibly histatin show greater adherence in the presence of TGM, their interactions with MUC7 may have allowed greater incorporation of this salivary mucin into the pellicle. MUC7 has been shown to form complexes with these proteins at its N-terminal region [46] and this might be a requirement for its incorporation into the pellicle in the oral cavity. Interestingly, this effect was not observed for MUC5B.

During the experiments, the decision was made, to complete the TGM assay by pre-incubation of saliva with TGM. This was done as previous work has shown TGM to be present in the saliva [7] which could alter protein binding due to cross-linking. However, TGM is also present on the mucosal epithelium [7,47] and it is possible that the mechanism of protein cross-linking is a result of that epithelial derived TGM.

## 5 Conclusion

Data from this paper demonstrates that salivary proteins have the ability to bind to multiple surface types. It is assumed that this flexibility is crucial to the formation of the salivary pellicle on all surfaces (hard and soft) within the oral cavity. It is likely that the oral mucosa is initially hydrophobic before the binding of salivary proteins, which then alter pellicle development through interactions with each other. Small proteins including statherin show strong interactions with hydrophobic particles, suggesting they act as “precursor” pellicle proteins, i.e. adsorbed first [27]. As MUC5B did not bind to any particle but is known to be part of the mucosal pellicle its lack of binding may be due to other factors such as the absence of membrane bound MUC1 [36], which may also aid in the initiation of the salivary pellicle development. Proteins such as MUC7 and IgA may form cross-links to enable incorporation into the mucosal pellicle whilst, some proteins may also become cross-linked through interactions of TGM. The use of particles with different surface chemistries has shown the unpredictable nature of protein binding from complex mixtures but may provide more useful insights into real biological phenomena.

## Figures and Tables

**Table 1:** Demonstrating the presence of specific proteins in protein elutions indicating the presence in pellicle on each particle surface from each type of saliva secretion. How proteins detected is described: CBB – Coomassie staining, PAS – PAS staining, WB – Western blotting and MPA – lectin binding using Western blotting. Symbols indicate presence (+) or absence (−) in the three elutions: SDS1, SDS2, boil elution (WMS only, not all proteins tested). * indicates that detection levels were too low to determine protein binding pattern (N/A indicates that the protein is not present in the saliva).

Saliva		WMS			PS			SMSL		
Particle	Detection method	PSt	MF +ve	Si −ve	PSt	MF +ve	Si −ve	PSt	MF +ve	Si −ve
MUC5B	PAS/WB	++−	−−−	++−	N/A			−−	−−	−−
MUC7	PAS	++−	−+−	+−−	N/A			−−	−−	++
gPRP (70 kDa)	MPA	−−−	−−−	+++	++	−−	++	N/A		
aPRP (28 kDa)	MPA	+++	−++	+++	++	−−	++	++	−−	++
bPRP PS ½ (44–48 kDa)	CBB	−−−	−−−	−−−	++	−−	++	N/A		
bPRP (10–20 kDa)	CBB	−−−	−−−	−−−	−−	−−	−−	N/A		
Agglutinin (600–700 kDa)	MPA	+−	−−	+−	+−	−−	++	+−	−−	?
Amylase (54 kDa)	CBB	−−−	−−−	−−−	−−	−−	−−	−−	−−	−−
CAVI	WB	+++	−−+	+−+	++	+−	+−	++	+−	++
Cystatin S	WB	+++	−++	−−+	N/A			++	−−	−−
Statherin	CBB	+++	−−+	−−−	++	+−	−−	++	−−	−−
Histatin (3–5 kDa)	CBB	*	−−	++	−−	−−	++	−−	−−	++
Secretory Component	CBB	++	++	+−	+−	+−	+−	++	++	++
Alpha-chain (IgA)	MPA	++	+−	+−	−−	−−	−+	++	+−	++
